# Interpretable machine learning identifies predictors of IGF-1 during growth hormone therapy in boys

**DOI:** 10.3389/fendo.2026.1873245

**Published:** 2026-10-16

**Authors:** Helena-Jamin Ly, Juulia T. Suvilehto, Beatrice Skyman, Carina Ankarberg-Lindgren, Hans Fors, Jesper Havsol, Jovanna Dahlgren

**Affiliations:** 1 Department of Pediatrics, Göteborg Pediatric Growth Research Center (GP-GRC), Institute of Clinical Sciences, Sahlgrenska Academy, University of Gothenburg, Gothenburg, Sweden; 2 Department of Pediatric Endocrinology, Sahlgrenska University Hospital, Queen Silvia Children’s Hospital, Gothenburg, Sweden; 3 AI Competence Center, Sahlgrenska University Hospital, Gothenburg, Sweden; 4 Medical Physics and Biomedical Engineering, Sahlgrenska University Hospital, Gothenburg, Sweden

**Keywords:** growth hormone therapy, IGF-1, machine learning, personalized medicine, prediction model

## Abstract

**Objectives:**

Insulin-like growth factor-1 (IGF-1) is widely used to guide growth hormone (GH) therapy, yet its predictors during the maintenance phase and puberty remain incompletely understood. We aimed to predict IGF-1 standard deviation scores (SDS) at 3 and 12 months in GH-treated boys and to identify key predictors of IGF-1 dynamics using interpretable modeling approaches.

**Methods:**

Data from 72 boys with growth hormone deficiency or idiopathic short stature from a previous Swedish clinical trial were analyzed using early-life characteristics, GH treatment variables, and puberty-related measures. Linear regression, Elastic Net regression, and explainable boosting machine (EBM) models were evaluated with nested five-fold patient-grouped cross-validation. Performance estimates were patient-balanced, with 95% confidence intervals based on 2,000 patient-cluster bootstrap resamples.

**Results:**

Elastic Net had the highest point estimates at 3 months, but confidence intervals did not establish a difference between the primary models. No clear differences between models were found at 12 months. Current IGF-1 SDS was the most prominent predictive feature, while additional treatment- and puberty-related variables were also selected across cross-validation models.

**Conclusion:**

Interpretable modeling approaches provided moderate predictive performance for future IGF-1 SDS during the maintenance phase and puberty. The findings indicate that prediction is informed by current IGF-1 SDS together with additional clinical variables, but further validation in independent cohorts is required before clinical application.

## 1 Introduction

Correct dosing of growth hormone (GH) in children with or without classic GH deficiency (GHD) can be challenging. Even after receiving treatment for several years, a significant percentage of patients fail to achieve target height based on parental heights, indicating that the effectiveness of treatment varies among individuals (1). GH dosing therefore requires individualized assessment and ongoing monitoring to optimize growth outcomes while minimizing risks. Insulin-like growth factor-1 (IGF-1)–based dosing, where the GH dose is titrated to achieve a desired IGF-1 standard deviation score (SDS), has been proposed as a strategy for personalizing treatment (2, 3). However, despite its widespread use in clinical practice, the predictors of IGF-1 levels during later stages of GH therapy remain incompletely understood.

IGF-1 levels are influenced by multiple physiological and treatment-related factors. In GH-naïve individuals, age and sex are known determinants, with IGF-1 levels increasing throughout childhood and reaching a peak during puberty (4–6). In prepubertal children, IGF-1 is associated with height velocity, whereas during puberty, IGF-1 levels may remain elevated despite declining growth velocity (4–6). Following the initiation of GH therapy, an initial catch-up phase characterized by rapid growth is typically followed by a maintenance phase and subsequently a pubertal growth spurt (2). During the early phase of treatment, IGF-1 appears to be positively correlated with GH dose (7, 8). In one of the few trials employing IGF-1-based dosing, Cohen et al. showed that higher GH doses were needed to maintain IGF-1 levels at the higher end of the normal range (9). Lundberg et al. showed that a higher GH dose led to a higher delta IGF-1 (ΔIGF-1) during both prepubertal and pubertal growth and resulted in a greater total gain in height (10). Conversely, Oberle et al. did not show any significant association between IGF-1 z-scores and GH dose in their cohort of prepubertal children on GH when adjusted for age, gender, race and insurance type (11).

During maintenance and pubertal growth, the dose dependency is not as obvious. Kim et al. demonstrated that changes in IGF-1 were weakly associated with treatment effects during the first 2 years of treatment, but they were no longer significant from the third year of treatment (12). This implies that other factors are more impactful at later treatment stages. For example, caloric or protein-restricted diets have been shown to decrease IGF-1 levels in both children and adults (13). Taken together, these findings indicate that IGF-1 dynamics during GH therapy reflect a complex interplay of physiological and treatment-related variables.

Most existing prediction models in pediatric GH therapy have focused on growth outcomes and have relied on multiple linear regression or empirical curve fitting approaches (14–17). More recently, machine learning methods have been applied to predict growth outcomes or treatment adherence (18, 19), while prediction of IGF-1 levels has received less attention, particularly during the later phases of growth hormone therapy where IGF-1 dynamics become more complex. Furthermore, while a model designed to predict growth response may maximize growth outcomes, it does not account for safety. This aspect of treatment can be addressed by focusing on IGF-1 as the predictive target. In non-GH-treated populations, both low and high IGF-1 levels are linked to mortality risk, underscoring the need for IGF-1 regulation in GH treatment (20).

Machine learning approaches are well suited to identifying complex and potentially non-linear relationships between clinical variables. Some models are so-called black-box models, where the reasoning behind the machine learning algorithms is difficult to understand. However, in healthcare settings, it is important that the clinicians are able to follow the model’s reasoning and evaluate the resulting recommendation (21); this is possible in so-called white-box models. In this study, we applied three interpretable modeling approaches with good performance in small datasets: linear regression, Elastic Net regression, and explainable boosting machine (EBM). Linear regression was used as a baseline method representing standard statistical analysis. Elastic Net regression combines L1 and L2 regularization to improve model stability and variable selection, particularly when multiple predictors are correlated (22, 23), while EBM is a tree-based gradient-boosting method designed to balance flexibility and interpretability (24–26). These approaches allow detailed examination of how individual predictors contribute to IGF-1 levels in complex datasets.

The aim of this study was to compare interpretable modeling approaches for the prediction of IGF-1 SDS and to identify key predictors of IGF-1 levels at 3 and 12 months during the maintenance growth phase and puberty in boys receiving growth hormone therapy.

## 2 Materials and methods

### 2.1 Study population

The study population originated from a larger cohort of 153 short-statured children who participated in the “Catch-up growth study” by Kriström et al. (27). Of these, 98 children who achieved catch-up growth were included in a subsequent clinical trial, the “Maintenance study”, a randomized, prospective, open-label study conducted in Sweden (study number NRA 6280003; ClinicalTrials.gov identifier NCT028779747) (28).

Children entering the Maintenance study had completed 2–3 years of GH treatment during the Catch-up growth study and were prepubertal. For children previously receiving individualized GH dosing, eligibility required an attained height SDS no more than 0.6 SDS below the mid-parental height SDS, whereas no corresponding auxological criterion was applied to the weight-based dose-control group (28). Of the 72 boys, 22 had GHD, defined as a peak GH concentration below 10 µg/L on both the arginine–insulin tolerance test and a 24-hour spontaneous GH profile, whereas the remaining 50 had idiopathic short stature (ISS).

Of the 98 participants in the Maintenance study, the present analysis was restricted to the 72 boys; the 26 girls were excluded because sex-related differences in hormonal regulation and growth patterns precluded combined modeling. Separate analysis cohorts were constructed for prediction of IGF-1 SDS at 3 and 12 months. After applying the prespecified eligibility criteria and requiring complete data for the outcome and the common set of 33 candidate predictors, 62 boys contributed 165 observation pairs to the 3-month analysis and 65 boys contributed 306 observation pairs to the 12-month analysis. Time points with 60 or more missed GH doses during the preceding year were excluded. Several participants contributed repeated observation pairs.

### 2.2 Data

The selected variables were based on previously identified predictors of growth (14). Early growth data were obtained from child welfare centers. Additional clinical data, including height, weight, testicular volume, IGF-1 levels, IGF-binding protein-3 (IGFBP3), and GH dose, were collected at least annually, and every three months during active participation in the Maintenance study.

Variables were categorized into three groups: early-life variables, variables related to the initiation of GH treatment, and variables corresponding to the time of prediction. Most participants contributed multiple data points (**Table 1**). Early-life and treatment-initiation variables remained constant within individuals, whereas prediction-time variables varied across time points. All variables included in the analysis are listed in **Supplementary Table S1**.

**Table 1:** Participant characteristics for the 3-month and 12-month prediction cohorts.

Variables	3-month prediction cohort	12-month prediction cohort
Subjects (n)	62	65
Datapoints (n)	165	306
Datapoints per subject (n)	2.0 [1.0,12.0]	5.0 [1.0,13.0]
Early life features		
Birth weight (g)	3253.3 (467.8)	3301.5 (469.5)
Birth length (cm)	49.0 [44.0,54.0]	49.0 [44.0,54.0]
Growth first year of life (cm)	23.0 [12.0,27.0]	23.0 [12.0,27.0]
Growth second year of life (cm)	9.2 (2.4)	9.1 (2.5)
Early treatment features		
Age at treatment start (years)	6.9 (2.1)	6.8 (2.0)
Stimulated GH peak (µg/L)	6.8 (3.8)	6.6 (3.7)
Height velocity one year before treatment (SDS)	-0.0 [-0.4,0.6]	-0.1 [-0.5,0.5]
Change in IGF-1 at 3 months on treatment (%)	121.4 [-16.1,598.4]	126.6 [-16.1,598.4]
Change in IGF-1 at 1 year on treatment (%)	182.5 [-16.2,817.6]	160.0 [-16.2,817.6]
Features at prediction time		
Age (years)	9.9 (2.3)	11.6 (2.4)
Weight (SDS)	-0.7 [-4.0,2.2]	-0.6 [-4.1,4.3]
Target height deficit (SDS)	-0.1 (0.6)	0.0 (0.8)
Change in target height deficit during the previous 12 months (SDS)	0.3 (0.2)	0.1 (0.3)
GH dose (mg/kg/day)	0.041 [0.016,0.101]	0.041 [0.017,0.101]
IGF-1 (SDS)	1.8 (1.2)	1.6 (1.1)
Testicular volume (mL)	2.0 [1.0,20.0]	3.0 [1.0,25.0]
Testosterone (nmol/L)	0.1 [0.0,29.3]	0.3 [0.0,31.2]
Estradiol (pmol/L)	1.0 [0.0,90.0]	4.0 [0.0,90.0]
Missing injections during the previous year (n)	2.0 [0.0,46.0]	2.0 [0.0,51.0]

Variables with normal distribution are presented as mean (SD), whereas variables with a non-normal distribution are presented as median [min,max].

Separate datasets were created for the two prediction horizons. For each index visit, candidate predictors represented information available at or before that visit, and the outcome was IGF-1 SDS measured approximately 3 or 12 months later. The nearest eligible IGF-1 measurement to the prespecified future date was used when it occurred within plus or minus 60 days. The same 33 candidate predictors and the same complete-case eligibility rule were used for all models within each prediction horizon.

### 2.3 Hormone analyses

Hormone analyses were conducted at the Gothenburg Pediatric Growth Research Centre Laboratory. Morning blood samples (08:00–10:00) for testosterone and estradiol were collected annually and stored at –80 °C. Sampling was performed at the onset of puberty, one and two years before, and up to three years after, allowing a maximum of six samples per participant.

Estradiol and testosterone were analyzed using validated high-sensitivity gas chromatography–tandem mass spectrometry (29). The lower limit of detection was 2 pmol/L (0.54 pg/mL) and 0.1 nmol/L (2.9 ng/dL) for estradiol and testosterone, respectively. For estradiol, the +total imprecision (CV) was 19% at 8 pmol/L (2.18 pg/mL) and 8% at ≥ 36 pmol/L (9.81 pg/mL). For testosterone, the total CV was 16% at 0.3 nmol/L (8.7 ng/dL) and< 10% at ≥ 1.5 nmol/L (43.3 ng/dL). Serum IGF-1 and IGFBP3 concentrations were analyzed using a specific radioimmunoassay (RIA) (Mediagnost GmbH, Tübingen, Germany) (30). For IGF-1, the total CV was 16% at 40 µg/L and 9% at ≥ 225 µg/L. For IGFBP3, the total CV was 10% independent of the concentration. The total imprecision CV for the IGF-1/IGFBP3 ratio was 17% at 2.1 and 15% at ≥ 5.0. Significant changes in hormonal variables were defined as $1.96\text{ x }\sqrt{2}\text{ x CVx}$, where CVx represents the coefficient of variation for the respective variable.

To address missing data, 31 IGF-1 and IGFBP3 analyses were performed on stored samples using the IDS iSYS assay (Immunodiagnostic Systems Holdings, Tyne and Wear, UK). As this method aligns well with the previous standard, no conversion was required (31). IGF-1 and IGFBP3 SDS were calculated using a healthy pediatric reference (5, 31).

### 2.4 Analysis cohort and validation strategy

For each prediction horizon, one common complete analysis cohort was used for all models. Model performance was evaluated using nested cross-validation with patient-level grouping. Participants were assigned to five reproducible outer folds using shuffled K-fold partitioning with random seed 2023. All observations from a participant were assigned to the same outer fold, and the identical outer-fold assignments were used for every model within a prediction horizon. In each outer iteration, models were developed using four folds and evaluated on the remaining fold, so that every eligible observation received one out-of-fold prediction from a model that had not been trained or tuned using that participant.

Hyperparameter selection was performed only within the corresponding outer training data using three-fold patient-grouped inner cross-validation. No participant occurred in both the training and validation subsets of an inner split, and the outer test fold was not used for scaling, feature selection, hyperparameter selection, or model fitting. The 3-month and 12-month analyses were performed separately.

### 2.5 Missing-data handling

Testosterone and estradiol values missing before the first measurement were assigned prepubertal baseline values corresponding to half the assay limit of detection; later values were not carried forward. During preprocessing, within-participant LOCF was applied to testicular volume, GH dose, target-height deficit SDS, and weight SDS. Rows with remaining missing model inputs were excluded. No LOCF-substituted values remained among the 165 observations in the 3-month cohort. At 12 months, one of 306 observations contained a carried-forward GH dose, and none contained LOCF values for the other variables. Hormone baseline substitutions and the complete imputation audit are reported in **Supplementary Table S3**.

### 2.6 Statistical analyses and modeling

Analyses were performed using Python 3.9.19, NumPy 1.23.3, pandas 2.2.2, scikit-learn 1.3.2, and InterpretML 0.6.2. The revised primary comparison included three interpretable regression approaches using the same 33 candidate predictors: full ordinary least-squares linear regression, Elastic Net regression, and an Explainable Boosting Machine (EBM). The full linear model was fitted without regularization. Predictor standardization was applied only for Elastic Net, and the outcome remained on its original IGF-1 SDS scale.

Elastic Net was implemented as a pipeline in which a StandardScaler and the Elastic Net estimator were fitted together within each training split, preventing information from inner validation or outer test participants from contributing to the scaling parameters. Hyperparameters were selected by exhaustive grid search over 20 logarithmically spaced alpha values from 10^-4 to 10^0.5 and L1 ratios of 0.10, 0.50, 0.70, 0.90, 0.95, 0.99, and 1.00, giving 140 candidate combinations. Random coordinate selection and random seed 2023 were used. Candidate models were ranked by root mean squared error in the three-fold patient-grouped inner cross-validation, and the selected pipeline was refitted on the complete outer training set.

EBM models were fitted using the InterpretML ExplainableBoostingRegressor. Within each outer training set, 100 candidate parameter configurations were evaluated by randomized search with random seed 2023 and three-fold patient-grouped inner cross-validation, using root mean squared error as the selection criterion. The prespecified distributions covered minimum leaf sizes of 2-3, learning rates from 0.001 to 0.501, cyclic progress from 0 to 1, smoothing rounds from 100 to 499, interaction allocation from 0 to 1, and greedy ratios from 0 to 4. The selected EBM was refitted on the complete outer training set before prediction of the outer fold.

Model performance was assessed using R^2^, MAE, and RMSE. Patient-balanced performance estimates were calculated from the pooled out-of-fold predictions, with each participant contributing equal total weight irrespective of the number of observations. Uncertainty was quantified using 2,000 patient-cluster bootstrap resamples, and performance estimates are reported with 95% confidence intervals.

Predictors with non-zero coefficients in all five outer-fold models were considered consistently selected predictive features. Coefficients were summarized as the median and range across the five outer-fold models.

The selected hyperparameters across the outer cross-validation folds are reported in **Supplementary Table S4**.

## 3 Results

Separate models were developed to predict 3-month and 12-month IGF-1 SDS. **Table 1** shows the participant characteristics for the 3-month and 12-month prediction cohorts.

### 3.1 Performance of models

Model performance for the prediction of 3- and 12-month IGF-1 SDS is presented in **Table 2**. Performance was assessed using the coefficient of determination (R^2^), mean absolute error (MAE), and root mean squared error (RMSE), with 95% confidence intervals. As a reference, a persistence model using current IGF-1 SDS as the prediction of future IGF-1 SDS showed lower predictive performance than the multivariable models at both prediction intervals. Results from additional comparison models are presented in **Supplementary Table S2**.

**Table 2:** Predictive performance of the primary models.

Prediction horizon	Model	R^2^ (95% CI)	MAE (95% CI)	RMSE (95% CI)
3 months	Full linear regression	0.50 (0.24 to 0.65)	0.68 (0.59 to 0.77)	0.83 (0.73 to 0.93)
3 months	Elastic Net	0.57 (0.41 to 0.67)	0.61 (0.52 to 0.71)	0.76 (0.66 to 0.86)
3 months	Explainable Boosting Machine	0.52 (0.35 to 0.63)	0.65 (0.56 to 0.74)	0.81 (0.69 to 0.92)
12 months	Full linear regression	0.45 (0.30 to 0.56)	0.67 (0.59 to 0.75)	0.85 (0.76 to 0.95)
12 months	Elastic Net	0.45 (0.33 to 0.55)	0.66 (0.60 to 0.73)	0.85 (0.75 to 0.96)
12 months	Explainable Boosting Machine	0.41 (0.30 to 0.50)	0.70 (0.63 to 0.77)	0.88 (0.78 to 0.99)

Values are point estimate (95% confidence interval). Higher R^2^ and lower MAE and RMSE indicate better predictive performance.

Estimates were calculated from five-fold patient-grouped out-of-fold predictions with equal total weight per participant. Percentile 95% confidence intervals were obtained from 2,000 patient-cluster bootstrap resamples.

MAE and RMSE are expressed in IGF-1 SDS units. CI, confidence interval; EBM, Explainable Boosting Machine; MAE, mean absolute error; RMSE, root mean squared error.

Patient-balanced performance estimates were calculated from pooled out-of-fold predictions.

### 3.2 Elastic net

Elastic Net identified five consistently selected predictive features at both prediction horizons, defined as predictors with non-zero coefficients across all five outer cross-validation models (**Figure 1**). For the 3-month prediction, these were IGF-1 SDS at prediction, IGF-1 increase, GH dose, testicular volume, and age at GH treatment start. For the 12-month prediction, the consistently selected predictive features were IGF-1 SDS at prediction, testosterone decrease, testicular volume, age at prediction, and IGF-1/IGFBP-3 increase. IGF-1 SDS at prediction showed the largest median coefficient at both prediction horizons.

**Figure 1:**
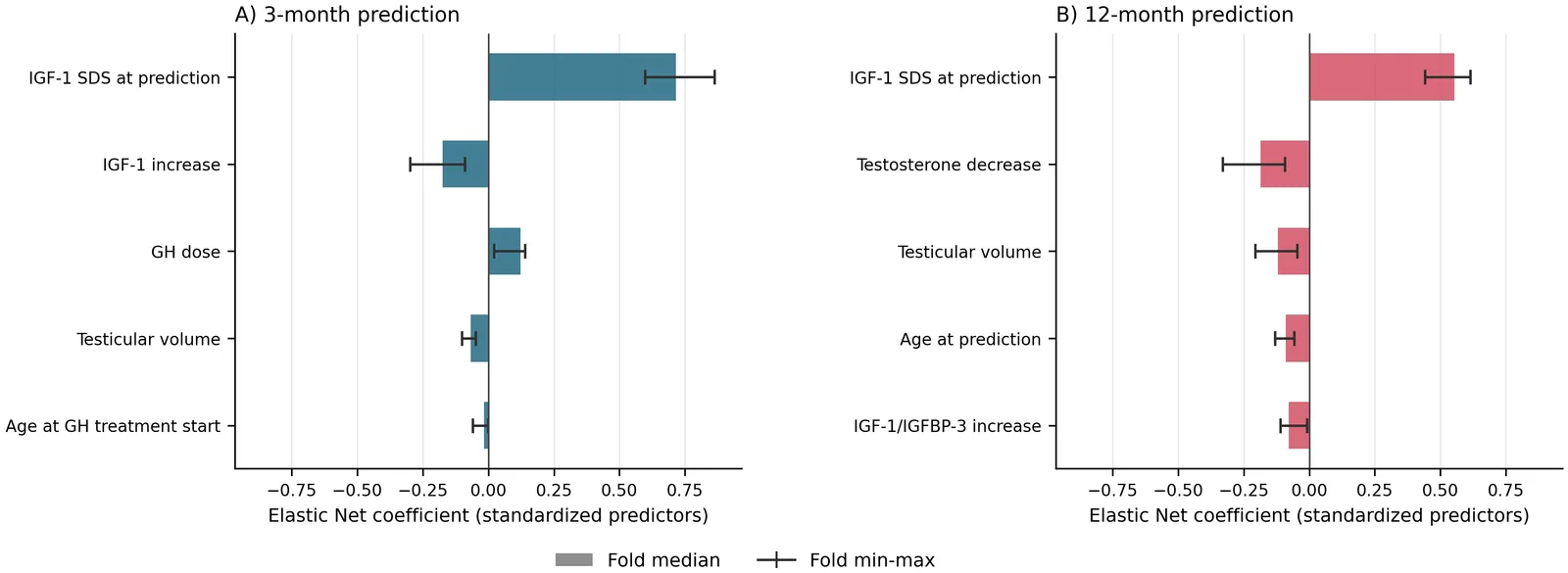
Stability of Elastic Net coefficients for 3- and 12-month IGF-1 SDS prediction. Panels **(A, B)** show predictors with non-zero coefficients in all five patient-grouped outer cross-validation models for the 3- and 12-month prediction horizons, respectively. Bars represent the median coefficient across the five models, and whiskers indicate the minimum and maximum coefficients. Predictors were standardized within each outer training set, whereas the outcome was not additionally scaled; coefficients therefore represent the change in predicted IGF-1 SDS per one-standard-deviation increase in the predictor. The whiskers describe variation across folds and are not confidence intervals.

### 3.3 Explainable boosting machine

EBM showed comparable predictive performance to the other models (**Table 2**). To assess the stability of feature importance, EBM main-effect importance was summarized across the five outer cross-validation models. IGF-1 SDS at prediction showed the highest median importance at both prediction horizons. The ten main effects with the highest median importance across the five models are presented in **Supplementary Figure S1**.

## 4 Discussion

Interpreting IGF-1 levels in the context of GH therapy is complex, as multiple physiological and treatment-related factors influence IGF-1 dynamics over time (32, 33). In this study, we compared conventional linear regression, Elastic Net regression, and EBM for prediction of IGF-1 SDS at 3 and 12 months during the maintenance phase and puberty. Elastic Net had the highest point estimates at 3 months, but confidence intervals did not establish a difference between the primary models. No clear differences between models were found at 12 months. EBM demonstrated comparable predictive performance and was included as a non-linear comparison model. The lower performance of the persistence benchmark further indicates that clinical variables beyond current IGF-1 SDS contribute additional information to the prediction of future IGF-1 levels.

The comparatively lower performance of ordinary linear regression may partly reflect the limitations of applying an unregularized linear model in a dataset with many potentially correlated predictors. Linear regression was included as a conventional and readily interpretable reference model against which other interpretable modeling approaches could be compared. Elastic Net may improve predictive performance in this setting by applying regularization and variable selection, thereby reducing the risk of overfitting. EBM provided a complementary assessment of predictive feature importance. Across the five cross-validation models, current IGF-1 SDS showed the highest median main-effect importance at both prediction horizons, consistent with its prominent role in the Elastic Net models. Several additional treatment- and puberty-related variables also showed predictive importance, although their importance varied across folds (**Supplementary Figure S1**). The greater flexibility of EBM did not result in clearly improved predictive performance compared with Elastic Net.

### 4.1 Growth hormone dose

GH dose received a non-zero Elastic Net coefficient in all five outer-fold models for the 3-month prediction. GH dose was also among the features with the highest median importance in the EBM analysis, supporting its relevance for short-term prediction of IGF-1 SDS. This is consistent with previous studies showing an association between GH dose and IGF-1 levels, although the strength of this relationship may vary across treatment phases and patient populations (9–12).

Unlike the initial catch-up phase, IGF-1 dynamics during the maintenance phase and puberty are influenced by multiple interacting factors, resulting in both increases and decreases in IGF-1 levels despite ongoing GH treatment.

### 4.2 Pubertal hormones

Puberty-related variables contributed particularly to the 12-month prediction. Testicular volume was consistently selected by Elastic Net at both prediction horizons, while age at prediction and testosterone decrease also received non-zero coefficients in all five outer-fold models at 12 months. In the EBM analysis, age, testicular volume, testosterone, and estradiol were among the more important features for the 12-month prediction. These findings support the relevance of pubertal development for IGF-1 dynamics during longer prediction intervals. Interpretation of IGF-1 SDS during puberty is challenging, as pubertal development and sex steroid levels are not fully accounted for by age-based reference values (33).

### 4.3 Previous prediction models

Previous prediction models in pediatric GH therapy have primarily focused on growth outcomes and have relied on linear or empirical approaches (14–17). More recent machine learning studies have similarly focused on growth response or treatment adherence (18, 19). In contrast, prediction of IGF-1 levels during the maintenance phase and puberty has received less attention. The present study extends previous work by demonstrating that interpretable modeling approaches can identify predictors of IGF-1 dynamics during these later phases of treatment.

Despite the relatively small cohort, the prospective longitudinal design, repeated measurements, and limited missing data allowed investigation of IGF-1 dynamics over several years of treatment. Five-fold patient-grouped cross-validation ensured that observations from the same participant were not distributed across training and evaluation data, while patient-cluster bootstrap confidence intervals were used to quantify uncertainty in model performance. However, the models were developed and evaluated within a single cohort. The limited sample size and absence of external validation restrict the generalizability of these findings, and further validation in independent cohorts is required (34).

Although the best-performing models explained approximately half of the observed variance in IGF-1 SDS, a substantial proportion remained unexplained, reflecting the complexity of IGF-1 dynamics during the maintenance phase and puberty. In current clinical practice, GH dose adjustments are not based solely on IGF-1 levels, but are interpreted together with growth response, pubertal development, treatment adherence, and other clinical factors. A prediction error of approximately 0.6-0.7 SDS would therefore currently limit the use of these models as stand-alone tools for dose adjustment.

The primary aim of this study was not to develop a dosing algorithm for clinical use, but to evaluate whether IGF-1 levels during the maintenance phase and puberty can be predicted and to identify predictors of IGF-1 levels over time. The present findings demonstrate that interpretable modeling approaches can explain a substantial proportion of the observed variation in IGF-1 levels during a treatment phase where regulation is influenced by multiple interacting factors. Further validation in larger cohorts will be required before clinical implementation can be considered.

Overall, these findings suggest that IGF-1 dynamics during GH therapy are influenced by multiple physiological and treatment-related factors. Interpretable modeling approaches achieved moderate predictive performance and identified several clinically relevant predictors of IGF-1 levels during the maintenance phase and puberty. While further validation is required, these results provide new insight into the prediction of IGF-1 levels during later phases of GH treatment and may support the future development of more individualized treatment strategies.

## 5 Conclusion

Interpretable modeling approaches achieved moderate predictive performance for IGF-1 standard deviation scores and identified consistently selected predictive features during the maintenance phase and puberty in boys receiving growth hormone therapy. Current IGF-1 SDS was the most prominent predictive feature, while additional treatment- and puberty-related variables were also selected across cross-validation models. While further validation in independent cohorts is required, the use of interpretable models provides valuable insight into predictors of IGF-1 levels and may support the future development of more individualized approaches to growth hormone treatment.

## Data Availability

The raw data supporting the conclusions of this article will be made available by the authors, without undue reservation.
