# Supplementary material for: Interpretable machine learning identifies predictors of IGF-1 during growth hormone therapy in boys
Source: Front Endocrinol (Lausanne). 2026 Oct 16;17:1873245. doi: 10.3389/fendo.2026.1873245 (PMC13719384; doi:10.3389/fendo.2026.1873245)
Supplement: Supplementary file 1 [file SupplementaryFile1.docx]

**Supplementary**

Supplementary Table S1

Values are presented as mean (SD), median [min, max], or n (%). Stable patient characteristics are summarized once per patient, whereas time-varying characteristics are summarized across complete-case data points.

| **Variables** | **3-month prediction cohort** | **12-month prediction cohort** |
| --- | --- | --- |
| Subjects (n) | 62 | 65 |
| Datapoints (n) | 165 | 306 |
| Datapoints per subject (n) | 2.0 [1.0,12.0] | 5.0 [1.0,13.0] |
| **Early life features** |  |  |
| Birth weight (g) | 3253.3 (467.8) | 3301.5 (469.5) |
| Birth length (cm) | 49.0 [44.0,54.0] | 49.0 [44.0,54.0] |
| Growth first year of life (cm) | 23.0 [12.0,27.0] | 23.0 [12.0,27.0] |
| Growth second year of life (cm) | 9.2 (2.4) | 9.1 (2.5) |
| **Early treatment features** |  |  |
| Age at treatment start (years) | 6.9 (2.1) | 6.8 (2.0) |
| Stimulated GH peak (µg/L) | 6.8 (3.8) | 6.6 (3.7) |
| Height velocity one year before treatment (SDS) | -0.0 (0.2) | -0.1 [-0.5,0.5] |
| Change in IGF-1 at 3 months on treatment (%) | 121.4 [-16.1,598.4] | 126.6 [-16.1,598.4] |
| Change in IGF-1 at 1 year on treatment (%) | 182.5 (146.1) | 160.0 [-16.2,817.6] |
| **Features at prediction time** |  |  |
| Age (years) | 9.9 (2.3) | 11.6 (2.4) |
| Weight (SDS) | -0.8 (1.0) | -0.6 [-4.1,4.3] |
| Change in weight during the previous 12 months (SDS) | 0.2 (0.3) | 0.1 [-0.9,1.8] |
| Target height deficit (SDS) | -0.1 (0.6) | 0.0 (0.8) |
| Change in target height deficit during the previous 12 months (SDS) | 0.3 (0.2) | 0.1 (0.3) |
| GH dose (mg/kg/day) | 0.041 [0.016,0.101] | 0.041 [0.017,0.101] |
| Change in GH dose during the previous 3 months (mg/kg/day) | -0.001 [-0.049,0.048] | 0.000 [-0.049,0.048] |
| IGF-1 (SDS) | 1.8 (1.2) | 1.6 (1.1) |
| IGF-1 increase (yes/no) | 40 (24.2) | 85 (27.8) |
| IGF-1 decrease (yes/no) | 12 (7.3) | 17 (5.6) |
| IGFBP-3 increase (yes/no) | 17 (10.3) | 38 (12.4) |
| IGFBP-3 decrease (yes/no) | 7 (4.2) | 8 (2.6) |
| IGF-1/IGFBP-3 ratio | 5.6 (1.9) | 6.5 (2.1) |
| IGF-1/IGFBP-3 ratio increase (yes/no) | 15 (9.1) | 37 (12.1) |
| IGF-1/IGFBP-3 ratio decrease (yes/no) | 4 (2.4) | 4 (1.3) |
| Testicular volume (mL) | 2.0 [1.0,20.0] | 3.0 [1.0,25.0] |
| Change in testicular volume during the previous 12 months (mL) | 0.0 [-1.0,8.0] | 1.0 [-2.0,11.0] |
| Testosterone (nmol/L) | 0.1 [0.0,29.3] | 0.3 [0.0,31.2] |
| Testosterone increase (yes/no) | 40 (24.2) | 169 (55.2) |
| Testosterone decrease (yes/no) | 1 (0.6) | 3 (1.0) |
| Estradiol (pmol/L) | 1.0 [0.0,90.0] | 4.0 [0.0,90.0] |
| Estradiol increase (yes/no) | 27 (16.4) | 129 (42.2) |
| Estradiol decrease (yes/no) | 1 (0.6) | 1 (0.3) |
| Missing injections during the previous year (n) | 2.0 [0.0,46.0] | 2.0 [0.0,51.0] |
| Target IGF-1 (SDS) | 1.8 (1.1) | 1.4 (1.2) |

Supplementary Table S2

Predictive performance of all evaluated models for the 3- and 12-month IGF-1 SDS prediction. Patient-balanced estimates were obtained from five-fold patient-grouped cross-validation. Values are presented as estimates (95% CI), with confidence intervals based on 2,000 patient-cluster bootstrap resamples. The persistence benchmark uses IGF-1 SDS at the prediction time directly as the prediction of future IGF-1 SDS, whereas the IGF-1-only linear regression uses IGF-1 SDS at the prediction time as its sole predictor.

| **3-month prediction horizon** | | | |
| --- | --- | --- | --- |
| **Model** | **R² (95% CI)** | **MAE (95% CI)** | **RMSE (95% CI)** |
| Persistence | 0.39 (-0.02 to 0.63) | 0.70 (0.60 to 0.82) | 0.91 (0.77 to 1.05) |
| IGF-1-only linear regression | 0.51 (0.28 to 0.64) | 0.66 (0.57 to 0.76) | 0.82 (0.72 to 0.92) |
| Full linear regression | 0.50 (0.24 to 0.65) | 0.68 (0.59 to 0.77) | 0.83 (0.73 to 0.93) |
| Elastic Net | 0.57 (0.41 to 0.67) | 0.61 (0.52 to 0.71) | 0.76 (0.66 to 0.86) |
| Explainable Boosting Machine | 0.52 (0.35 to 0.63) | 0.65 (0.56 to 0.74) | 0.81 (0.69 to 0.92) |
| Symbolic regression | 0.54 (0.33 to 0.66) | 0.64 (0.56 to 0.73) | 0.79 (0.70 to 0.89) |
|  |  |  |  |
| **12-month prediction horizon** | | | |
| **Model** | **R² (95% CI)** | **MAE (95% CI)** | **RMSE (95% CI)** |
| Persistence | 0.27 (0.08 to 0.42) | 0.77 (0.68 to 0.85) | 0.98 (0.86 to 1.11) |
| IGF-1-only linear regression | 0.38 (0.25 to 0.49) | 0.70 (0.62 to 0.77) | 0.90 (0.79 to 1.03) |
| Full linear regression | 0.45 (0.30 to 0.56) | 0.67 (0.59 to 0.75) | 0.85 (0.76 to 0.95) |
| Elastic Net | 0.45 (0.33 to 0.55) | 0.66 (0.60 to 0.73) | 0.85 (0.75 to 0.96) |
| Explainable Boosting Machine | 0.41 (0.30 to 0.50) | 0.70 (0.63 to 0.77) | 0.88 (0.78 to 0.99) |
| Symbolic regression | 0.34 (0.22 to 0.45) | 0.73 (0.65 to 0.81) | 0.93 (0.82 to 1.06) |

| Supplementary Table S3  Missing-data substitution audit. Substituted values retained in the final 3- and 12-month analysis cohorts. | | | | | |
| --- | --- | --- | --- | --- | --- |
|  |  |  |  |  |  |
| **Variable** | **Substitution method** | **3-month values** | **3-month participants** | **12-month values** | **12-month participants** |
| Testosterone | Pre-first-measurement substitution with 0.05 nmol/L (half the assay limit of detection) | 107/165 (64.8%) | 38/62 (61.3%) | 81/306 (26.5%) | 37/65 (56.9%) |
| Estradiol | Pre-first-measurement substitution with 1 pmol/L (half the assay limit of detection) | 107/165 (64.8%) | 38/62 (61.3%) | 81/306 (26.5%) | 37/65 (56.9%) |
| Testicular volume | Within-participant last observation carried forward (LOCF) | 0/165 (0.0%) | 0/62 (0.0%) | 0/306 (0.0%) | 0/65 (0.0%) |
| GH dose per body weight | Within-participant last observation carried forward (LOCF) | 0/165 (0.0%) | 0/62 (0.0%) | 1/306 (0.3%) | 1/65 (1.5%) |
| Target-height deficit SDS | Within-participant last observation carried forward (LOCF) | 0/165 (0.0%) | 0/62 (0.0%) | 0/306 (0.0%) | 0/65 (0.0%) |
| Weight SDS | Within-participant last observation carried forward (LOCF) | 0/165 (0.0%) | 0/62 (0.0%) | 0/306 (0.0%) | 0/65 (0.0%) |
|  |  |  |  |  |  |
| Values are n/N (%), using all analysis observations or all participants in the corresponding final cohort as the denominator. | | | | | |
| *Final cohorts: 3 months, 62 participants and 165 observations; 12 months, 65 participants and 306 observations. Counts are variable-specific and are not mutually exclusive.* | | | | | |
| *LOCF, last observation carried forward; GH, growth hormone; SDS, standard deviation score.* | | | | | |

Supplementary Table S4

Hyperparameter tuning and selected configurations. Searches were repeated independently within each outer training fold. Selected values are from the final 3- and 12-month reanalysis runs.

| **Panel A. Validation design and hyperparameter search spaces** | | | | | | | | | | | | | |  |  |  |  |  |  |
| --- | --- | --- | --- | --- | --- | --- | --- | --- | --- | --- | --- | --- | --- | --- | --- | --- | --- | --- | --- |
| **Model** | | **Parameter** | | | | **Search space or setting** | | | **Implementation note** | | | | |  |  |  |  |  |  |
| Common | | Candidate predictors | | | | 33 predictors | | | Identical predictor set for all three primary models | | | | |  |  |  |  |  |  |
| Common | | Outer validation | | | | 5 patient-grouped folds | | | Each participant contributed to one outer test fold | | | | |  |  |  |  |  |  |
| Common | | Inner tuning | | | | 3 patient-grouped folds | | | Patients were shuffled with seed 2023 and kept intact | | | | |  |  |  |  |  |  |
| Common | | Selection metric | | | | Root mean squared error (RMSE) | | | Implemented as neg_root_mean_squared_error | | | | |  |  |  |  |  |  |
| Common | | Refitting | | | | Best configuration refitted | | | Refitted on the complete outer training set | | | | |  |  |  |  |  |  |
| Elastic Net | | Preprocessing | | | | StandardScaler within pipeline | | | Fitted in each training split; outcome not scaled | | | | |  |  |  |  |  |  |
| Elastic Net | | Search | | | | Exhaustive grid search | | | 140 combinations per outer fold | | | | |  |  |  |  |  |  |
| Elastic Net | | alpha | | | | 20 log-spaced values: 10^-4 to 10^0.5 | | | Endpoints included; numpy.logspace(-4, 0.5, 20) | | | | |  |  |  |  |  |  |
| Elastic Net | | L1 ratio | | | | 0.10, 0.50, 0.70, 0.90, 0.95, 0.99, 1.00 | | | 1.00 corresponds to Lasso | | | | |  |  |  |  |  |  |
| Elastic Net | | Coordinate selection | | | | random | | | Fixed for all candidates | | | | |  |  |  |  |  |  |
| Elastic Net | | Random seed | | | | 2023 | | | Elastic Net estimator and patient-level fold construction | | | | |  |  |  |  |  |  |
| EBM | | Estimator | | | | ExplainableBoostingRegressor | | | n_jobs = 1 | | | | |  |  |  |  |  |  |
| EBM | | Search | | | | Randomized search | | | 100 sampled configurations per outer fold | | | | |  |  |  |  |  |  |
| EBM | | min_samples_leaf | | | | Discrete uniform: {2, 3} | | | scipy.stats.randint(2, 4) | | | | |  |  |  |  |  |  |
| EBM | | learning_rate | | | | Uniform: [0.001, 0.501) | | | scipy.stats.uniform(0.001, 0.5) | | | | |  |  |  |  |  |  |
| EBM | | cyclic_progress | | | | Uniform: [0, 1) | | | scipy.stats.uniform(0, 1) | | | | |  |  |  |  |  |  |
| EBM | | smoothing_rounds | | | | Discrete uniform: 100-499 | | | scipy.stats.randint(100, 500) | | | | |  |  |  |  |  |  |
| EBM | | interactions | | | | Uniform: [0, 1) | | | scipy.stats.uniform(0, 1) | | | | |  |  |  |  |  |  |
| EBM | | greedy_ratio | | | | Uniform: [0, 4) | | | scipy.stats.uniform(0, 4) | | | | |  |  |  |  |  |  |
| EBM | | Random seed | | | | 2023 | | | Estimator, randomized search, and patient-level folds | | | | |  |  |  |  |  |  |
|  | |  | | | |  | | |  | | | | |  |  |  |  |  |  |
| **Panel B. Elastic Net parameters selected within each outer fold** | | | | | | | | | | | | | |  |  |  |  |  |  |
| **Prediction horizon** | | **Outer fold** | | **alpha** | | | **L1 ratio** | | | **Non-zero coefficients** | | **Mean inner-validation RMSE (tuning only)** | |  | | |  |  | |
| 3 months | | 1 | | 0.069519 | | | 1.000000 | | | 8 | | 0.782 | |  | | |  |  | |
| 3 months | | 2 | | 0.069519 | | | 1.000000 | | | 11 | | 0.712 | |  | | |  |  | |
| 3 months | | 3 | | 0.119935 | | | 0.950000 | | | 10 | | 0.851 | |  | | |  |  | |
| 3 months | | 4 | | 0.069519 | | | 1.000000 | | | 13 | | 0.786 | |  | | |  |  | |
| 3 months | | 5 | | 0.069519 | | | 0.900000 | | | 13 | | 0.764 | |  | | |  |  | |
| 12 months | | 1 | | 0.069519 | | | 1.000000 | | | 13 | | 0.935 | |  | | |  |  | |
| 12 months | | 2 | | 0.119935 | | | 1.000000 | | | 5 | | 0.832 | |  | | |  |  | |
| 12 months | | 3 | | 0.119935 | | | 1.000000 | | | 7 | | 0.893 | |  | | |  |  | |
| 12 months | | 4 | | 0.013539 | | | 0.950000 | | | 25 | | 0.955 | |  | | |  |  | |
| 12 months | | 5 | | 0.069519 | | | 1.000000 | | | 11 | | 0.884 | |  | | |  |  | |
|  | |  | |  | | |  | | |  | |  | |  | | |  |  |  |
| **Panel C. EBM parameters selected within each outer fold** | | | | | | | | | | | | | |  |  |  |  |  |  |
| **Prediction horizon**  **(months)** | **Outer fold** | | **Min. leaf size** | | **Learning rate** | | **Cyclic progress** | **Smoothing rounds** | | | **Interactions** | **Greedy ratio** | **Mean inner-validation RMSE (tuning only)** |  |  |  |  |  |  |
| 3 | 1 | | 3 | | 0.487698 | | 0.515397 | 226 | | | 0.925614 | 1.330493 | 0.870773 |  |  |  |  |  |  |
| 3 | 2 | | 3 | | 0.406169 | | 0.770764 | 471 | | | 0.791621 | 2.386802 | 0.818596 |  |  |  |  |  |  |
| 3 | 3 | | 3 | | 0.457396 | | 0.863313 | 136 | | | 0.110048 | 1.566881 | 0.905532 |  |  |  |  |  |  |
| 3 | 4 | | 2 | | 0.486871 | | 0.347107 | 489 | | | 0.708877 | 3.587395 | 0.864716 |  |  |  |  |  |  |
| 3 | 5 | | 3 | | 0.466266 | | 0.454927 | 344 | | | 0.378525 | 0.783455 | 0.862899 |  |  |  |  |  |  |
| 12 | 1 | | 3 | | 0.328545 | | 0.694868 | 373 | | | 0.873939 | 1.725906 | 0.940454 |  |  |  |  |  |  |
| 12 | 2 | | 3 | | 0.487698 | | 0.515397 | 226 | | | 0.925614 | 1.330493 | 0.862640 |  |  |  |  |  |  |
| 12 | 3 | | 3 | | 0.457396 | | 0.863313 | 136 | | | 0.110048 | 1.566881 | 0.934360 |  |  |  |  |  |  |
| 12 | 4 | | 2 | | 0.343523 | | 0.835714 | 464 | | | 0.671624 | 0.350606 | 0.956515 |  |  |  |  |  |  |
| 12 | 5 | | 3 | | 0.457396 | | 0.863313 | 136 | | | 0.110048 | 1.566881 | 0.925444 |  |  |  |  |  |  |
|  |  | |  | |  | |  |  | | |  |  |  |  |  |  |  |  |  |
| *Abbreviations: EBM, Explainable Boosting Machine; CV, cross-validation; RMSE, root mean squared error.* | | | | | | | | | | | | | |  |  |  |  |  |  |
| *Continuous EBM intervals follow scipy.stats parameterization; the upper endpoint is not included.* | | | | | | | | | | | | | |  |  |  |  |  |  |
| *Source: growthcurves/modeling/reanalysis_baselines.py, growthcurves/modeling/reanalysis_ebm.py, params.yaml, and final selected-parameter reports.* | | | | | | | | | | | | | |  |  |  |  |  |  |

Supplementary Figure S1

**Stability of EBM main-effect importance for 3- and 12-month IGF-1 SDS prediction.** Panels A and B show the ten main effects with the highest median importance across the five patient-grouped outer cross-validation models for the 3- and 12-month prediction horizons, respectively. Bars represent the median mean absolute EBM term score, and whiskers indicate the minimum and maximum across the five models. The scores describe the magnitude of each term’s contribution to prediction but do not indicate effect direction, causality, or an independently adjusted association. Whiskers show descriptive fold-to-fold variation and are not confidence intervals. Interaction terms were excluded because the fitted interaction structures differed across outer folds.
